# Physician referral patterns and racial disparities in total hip replacement: A network analysis approach

**DOI:** 10.1371/journal.pone.0193014

**Published:** 2018-02-20

**Authors:** Hassan M. K. Ghomrawi, Russell J. Funk, Michael L. Parks, Jason Owen-Smith, John M. Hollingsworth

**Affiliations:** 1 Departments of Surgery and Pediatrics, Feinberg School of Medicine, Northwestern University, Chicago, Illinois, United States of America; 2 Center for Healthcare Studies, Feinberg School of Medicine, Northwestern University, Chicago, Illinois, United States of America; 3 Carlson School of Management, University of Minnesota, Minneapolis, Minnesota, United States of America; 4 Department of Orthopedic Surgery, Hospital for Special Surgery, New York City, New York, United States of America; 5 Department of Sociology and Institute for Research on Innovation and Science, University of Michigan, Ann Arbor, Michigan, United States of America; 6 Department of Urology, University of Michigan, Ann Arbor, Michigan, United States of America; University of Sydney, AUSTRALIA

## Abstract

**Background:**

Efforts to reduce racial disparities in total hip replacement (THR) have focused mainly on patient behaviors. While these efforts are no doubt important, they ignore the potentially important role of provider- and system-level factors, which may be easier to modify. We aimed to determine whether the patterns of interaction among physicians around THR episodes differ in communities with low versus high concentrations of black residents.

**Materials and methods:**

We analyzed national Medicare claims from 2008 to 2011, identifying all fee-for-service beneficiaries who underwent THR. Based on physician encounter data, we then mapped the physician referral networks at the hospitals where beneficiaries’ procedures were performed. Next, we measured two structural properties of these networks that could affect care coordination and information sharing: clustering, and the number of external ties. Finally, we estimated multivariate regression models to determine the relationship between the concentration of black residents in the community [as measured by the hospital service area (HSA)] served by a given network and each of these 2 network properties.

**Results:**

Our sample included 336,506 beneficiaries (mean age 76.3 ± SD), 63.1% of whom were women. HSAs with higher concentrations of black residents tended to be more impoverished than those with lower concentrations. While HSAs with higher concentrations of black residents had, on average, more acute care beds and medical specialists, they had fewer surgeons per capita than those with lower concentrations. After adjusting for these differences, we found that HSAs with higher concentrations of black residents were served by physician referral networks that had significantly higher within-network clustering but fewer external ties.

**Conclusions:**

We observed differences in the patterns of interaction among physicians around THR episodes in communities with low versus high concentrations of black residents. Studies investigating the impact of these differences on access to quality providers and on THR outcomes are needed.

## Introduction

Over half a million total hip replacements (THRs) are performed each year. Not only is THR a common procedure, but it is also highly effective for treatment of end-stage hip osteoarthritis (OA) [1]. Despite this, large racial disparities exist in its utilization. In particular, black patients are treated with THR at a 50% lower rate than whites in the Medicare population, and this difference in utilization has persisted for over two decades [2, 3]. Moreover, when black patients do undergo THR, the procedure is more likely to be performed at a low-volume hospital where outcomes are generally worse.

The underlying causes of these racial disparities are complex and not well understood [4]. To date, most research has focused on patient-level factors. For example, studies indicate that black patients may be less willing to undergo THR because of widespread beliefs that OA is a normal part of aging. Data also suggest that black patients are skeptical about the procedure’s effectiveness and that they have concerns about its safety [4, 5]. As such, efforts to address racial disparities have focused mainly on changing black patients’ behaviors through educating them about the benefits of THR [6]. While these efforts are no doubt important, there is increasing interest in the potential role of provider- and health system-level factors, which may be easier to modify. More specifically, researchers have begun investigating the influence that physician referral networks have on patient access to surgical care [7–11].

Older patients with OA are usually managed by their primary care physician (PCP) until they reach end-stage disease [12], at which point they are referred to an orthopedic surgeon for definitive treatment [10]. Seminal work by Bach and colleagues showed that the PCPs who predominantly care for black patients face greater difficulties in referring to high-quality surgical specialists, obtaining necessary diagnostic imaging, and starting the nonemergency admission process than physicians who treat white patients [13]. In this way, the referral networks in which these physicians are embedded could adversely affect their patients’ surgical outcomes. To explore this possibility, we examined the association between the racial composition of a community and the characteristics of the physician referral networks formed around THR procedures in these communities.

## Materials and methods

### Data source and study population

For our study, we used Medicare data to construct physician referral networks for patients undergoing THR. In brief, a network is a collection of points (referred to as nodes) that are connected in pairs by lines (or ties). In the referral networks described below, the nodes represent individual physicians who provided care around a surgical episode, and the ties represent patients shared between two physicians. Prior empirical studies have shown that medical claims can be used to identify meaningful referral relationships between physicians [8].

To begin, we used appropriate *International Classification of Diseases*, *Ninth Revision*, procedure codes to identify all Medicare beneficiaries aged 66 years and older who underwent THR between January 1, 2008 and December 30, 2011, as well as the hospital where their surgery was performed, from the Medicare Provider Analysis and Review (MedPAR) file. For inclusion, we required beneficiaries to have continuous enrollment in Parts A and B for six months prior to their index admission and 60 days after discharge. We excluded Medicare Advantage beneficiaries since healthcare services delivered to them are inconsistently captured in their claims.

### Mapping physician referral networks

Once all eligible beneficiaries were identified, we determined which physicians provided care to them during their surgical episodes. To do this, we first constructed a claims window around their index hospitalization that began 30 days prior to admission and extended 60 days after discharge. Next, we distinguished all physicians (based on unique National Provider Identifiers and Medicare Specialty Codes) who billed for services on the beneficiaries’ behalf during this window. We included claims from the treating surgeon (i.e., the surgeon who billed for a THR procedure closest to the beneficiary’s surgery date), PCPs, and medical and other surgical specialists. We excluded claims from physicians who are not involved in direct patient care (e.g., radiology) or who have limited roles in perioperative management (e.g., anesthesia).

After all physicians participating in each surgical episode were identified, we aggregated across a hospital’s discharges in a given calendar year to create the bipartite (or two-mode) physician referral network in a local health system. In bipartite networks, nodes of the same class are never directly connected to each other. Because our research question centered on relationships between physicians, we created a unipartite projection of the bipartite networks such that physicians were connected directly and ties between them were weighted by the number of shared patients.

### Characterizing physician referral networks

Next, we used network analytical tools to characterize the level of care coordination and information sharing among physicians within these networks. We studied two network characteristics that, based on the work of Bach et al. (13), may lie in the causal pathway of the observed THR racial disparities: 1) the network’s clustering coefficient, and 2) its number of external ties. Clustering refers to the tendency for physicians in a network to assemble into tightly interconnected groups (referred to as cliques) around shared patients. Members of highly clustered networks share a wealth of information about common patients. However, they may suffer isolation because clustered networks operate with a high level of independence from their surroundings [14]. External ties represent the tendency for physicians to have contact with practitioners outside of their immediate area. External ties enhance a network as conduits for novel knowledge exchange with practitioners outside of the network [15]. When they are expressed in the form of shared patients, these ties may also indicate ability of network physicians to refer their patients to facilities outside their network when needed. We calculated each network’s clustering as the probability that two physicians in the network—each of whom shared a patient with a common third doctor—also shared a patient themselves. We calculated the number of external ties as the total physicians in a given network who practiced outside the Core-Based Statistical Area (CBSA) where the hospital anchoring it was located.

### Measuring the racial composition of the community served by a physician referral network

Next, we determined the concentration of black residents in the community served by each physician referral network. We used the hospital service area (HSA) boundaries, as described in the Dartmouth Atlas (www.dartmouthatlas.org), to define the community. An HSA is a collection of ZIP codes whose residents receive most of their healthcare from the hospitals in that area. We used publically available data from the 2010 U.S. Census to determine the concentration of black residents by dividing the number of black residents in an HSA by the total number of residents in it based on population estimates from the ZIP Code Tabulation Area Demographic Profile Summary File. We then sorted HSAs by their percentage of black residents into three comparably sized tertiles of low (0% to 2%), moderate (2% to 11%), and high (11% to 80%) concentration.

### Statistical analysis

Using the network-year as our unit of analysis, we compared the HSAs served by physician referral networks with one-way analysis of variance across a variety of sociocultural and healthcare capacity factors, stratifying by the concentration of black residents. Sociocultural factors included the concentration of residents in each HSA living below the federal poverty line, the concentration living in rural areas, the concentration holding a graduate or professional degree, and the concentration over age 65. We also assessed differences across HSAs with respect to their ethnic composition by developing measures of their total population and their concentration of Hispanic residents. Healthcare capacity factors that we examined included the number of acute care beds, PCPs, specialist physicians, and surgeons per capita (obtained from the *Dartmouth Atlas*). We also compared the hospitals anchoring our networks by the average Charlson index score for their patients, their mean annual volume, the number of referrals that they received from outside the HSA where the hospital was located, the proportion of severe complications within 60 days, and the hospital’s academic affiliation.

We then applied multivariate regression to determine whether the patterns of connection among physicians in a network were associated with the concentration of black residents in the HSA that the network served. We ran separate models with each network property as the dependent variable. Because clustering is a concentration, we used a random-effects tobit specification with left and right censoring at 0 and 1, respectively. We used a random-effects negative binomial specification to model external ties, as the variable is a count and takes on only non-negative integer values. The main independent variable was the concentration of black residents in the HSA served by the network. Our models were adjusted for sociocultural, healthcare capacity, and hospital factors described above. Finally, we controlled for the number of physicians in the network.

We performed all analyses using Stata SE Version 13.1. All tests were two-tailed and we set the probability of type 1 error at 0.05. The institutional review board at our institution approved this study.

## Results

In total, our study population included 336,506 beneficiaries, who underwent THR procedures performed at 3,405 hospitals over the study interval. The average patient age was 76.3 (SD 6.9). 63.1% of patients were female, and 3.3% were black. Table 1 displays differences between HSAs with low, moderate, and high black resident concentrations. There were statistically significant differences across all sociocultural and healthcare capacity factors. Specifically, there were higher concentrations of residents under poverty line, higher numbers of acute beds, higher numbers of medical specialists, and lower numbers of surgeons per capita in HSAs with high concentrations of African Americans. There were also statistically significant differences across most anchor hospital measures. In particular, anchor hospitals in communities with a high concentration of black residents tended to have higher mean Charlson scores and complication rates. These hospitals were more likely to be affiliated with an academic institution, as well.

**Table 1:** Characteristics of HSAs and anchor hospitals in 2011, stratified by the concentration of black residents.

	Low concentration		Moderate concentration		High concentration		
	Mean	SD	Mean	SD	Mean	SD	P-Value
**HSA-level**							
*Sociocultural measures*							
Population (log)	11.16	1.20	12.47	1.32	13.12	1.39	0.00
Concentration of Hispanic residents	0.10	0.15	0.15	0.16	0.13	0.13	0.00
Concentration of residents with graduate education	0.09	0.05	0.11	0.06	0.10	0.05	0.00
Concentration of residents living beneath the federal poverty line	0.13	0.05	0.14	0.05	0.18	0.05	0.00
Concentration of residents living in rural areas	0.40	0.26	0.19	0.20	0.16	0.20	0.00
Concentration of residents aged 65 and over	0.16	0.04	0.14	0.04	0.13	0.03	0.00
*Healthcare capacity measures*							
Acute care hospital beds per 1,000 residents	2.45	0.81	2.36	0.73	2.71	0.74	0.00
PCPs per 100,000 residents	77.38	21.92	68.52	16.04	69.54	16.88	0.00
Medical specialists per 100,000 residents	37.59	12.09	41.70	12.81	47.66	15.27	0.00
Surgeons per 100,000 residents	47.05	13.98	41.21	11.26	40.93	9.59	0.00
**Hospital-level**							
Number of patients	17.20	21.96	28.40	41.52	28.77	37.49	0.00
Number of physicians (log)	3.19	1.04	3.89	1.02	3.88	1.13	0.00
Concentration of patients from outside the CBSA	0.38	0.29	0.40	0.25	0.40	0.28	0.24
Academic hospital	0.20	0.40	0.34	0.47	0.41	0.49	0.00
Charlson score	0.98	0.71	1.15	0.68	1.28	0.94	0.00
Concentration of patients living below federal poverty line†	0.13	0.05	0.13	0.05	0.15	0.06	0.00
Concentration of patients with graduate education†	0.08	0.04	0.10	0.05	0.10	0.05	0.00
Concentration of patients living in a rural area †	0.45	0.27	0.27	0.23	0.25	0.24	0.00
Concentration of Hispanic patients †	0.09	0.14	0.13	0.14	0.10	0.11	0.00
Concentration of black patients	0.00	0.02	0.02	0.05	0.10	0.17	0.00
Hospitals (N)	1005		1006		1002		

**Abbreviations:** CBSA, core based statistical area; Coeff, coefficient; d. f., degrees of freedom; HSA, hospital service area; PCP, primary care physician; S. D., standard deviation.

Note: All tests are two-tailed tests (one-way ANOVA). Data on sociocultural measures (ex: total resident population, race/ethnicity measures, rural/urban designation, poverty, and education) were compiled using data from the 2010 U. S. Census, then aggregated from the ZIP Code Tabulation Area level to the HSA level by matching local ZIP codes. Capacity and some hospital-level measures (e. g., academic affiliation) were compiled using statistics from the Dartmouth Atlas of Health Care and the American Hospital Association Annual Survey. Other measures (ex: total patients/physicians) were calculated using the Medicare Provider Analysis and Review data for 2008 to 2011 hip replacement procedures.

^†^ Estimated using levels found in patients’ home zip codes (low concentration range 0.00–0.02; moderate concentration range 0.03–0.11, high concentration range 0.12–0.80).

**Fig 1:**
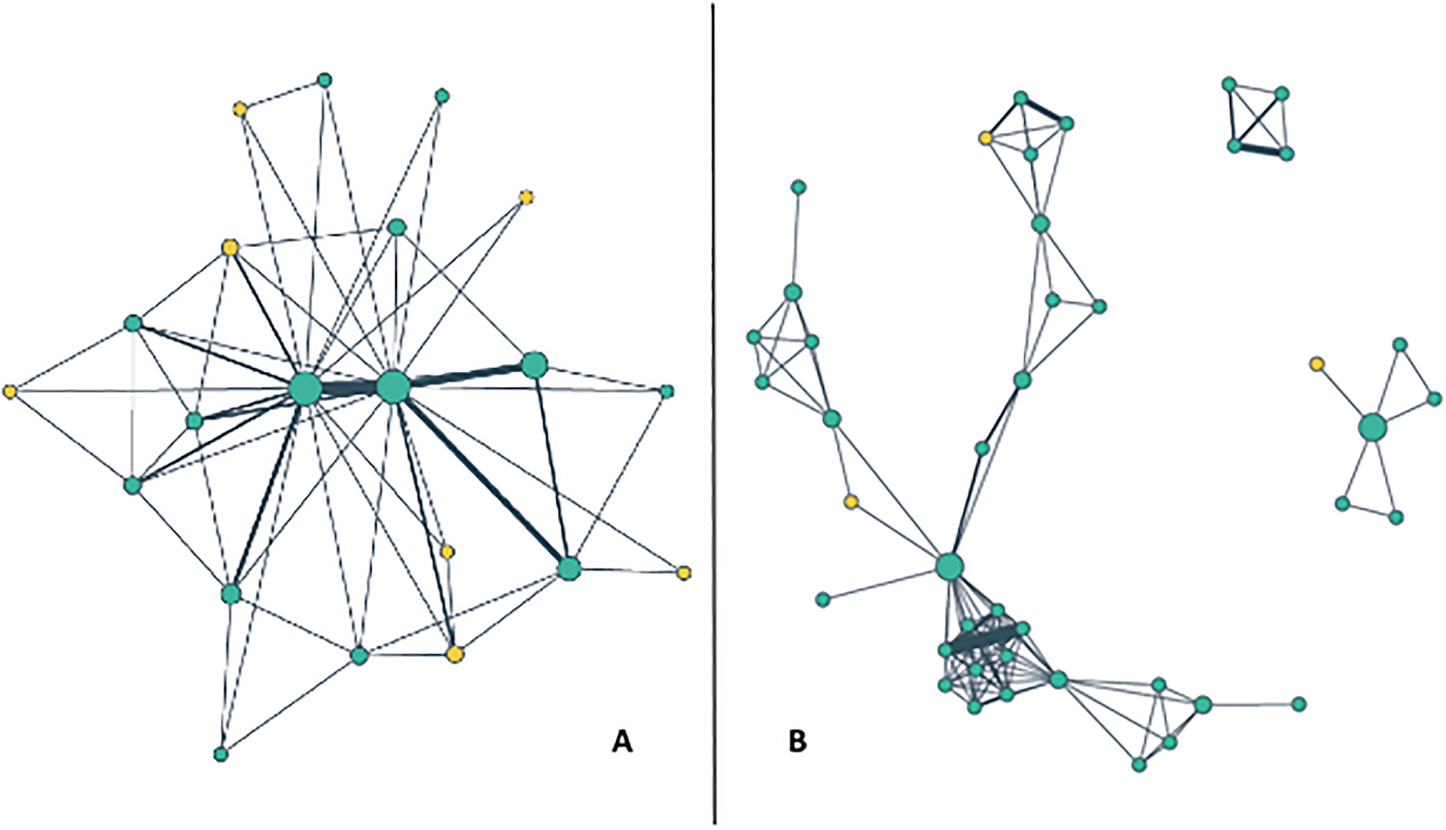
Physician networks in 2 California communities in 2011 with low (A) vs. high (B) concentration of blacks. (A) Hospital serving fewer black residents in HSA, % black = 0.00, clustering coefficient = 0.39, external ties = 7. (B) Hospital serving more black residents in HSA, % black = 0.13, clustering coefficient = 0.82, external ties = 3.

On multivariate analysis, physician referral networks serving HSAs with high concentrations of black residents differed in a number of ways from those serving HSAs with low concentrations of black residents (Table 2). After adjusting for the HSA- and hospital-level factors described above, physicians in networks serving HSAs with high concentrations of black residents were more likely to cluster in highly interconnected groups (coefficient = 0.08, p<0.01) compared to those in networks serving HSAs with low concentrations of black residents. In addition, networks serving HSAs with high concentrations of black residents were less likely to have external ties (coefficient = -1.92, p<0.001) compared to physicians practicing in HSAs with lower concentrations of black residents. Fig 1 visually illustrates these differences in the physician networks of two California hospitals, where green dots represent network physicians and yellow dots represent physicians outside of the CBSA. Fig 1A, which shows how physician network at a hospital serving a community with low concentration of black residents, reveals low clustering and 7 external ties. Fig 1B, which shows a physician network at a hospital serving a community with high concentration of black residents, reveals high clustering of 3 separate groups and 3 external ties, with the largest group having two external ties, and another group having no external ties.

**Table 2:** Multivariate models of physician networks and concentration of black residents in U. S. hospitals.

	Model 1		Model 2	
	Clustering		External ties	
	Coef.	S. E.	Coef.	S. E.
Concentration of black residents	0.08**	0.03	-1.92***	0.25
**HSA-level**				
*Sociocultural measures*				
Population (log)	0.00	0.00	-0.10***	0.02
Concentration of Hispanic residents	-0.05	0.04	-1.17***	0.27
Concentration of residents with graduate education	0.09	0.07	-0.77	0.53
Concentration of residents living beneath the federal poverty line	-0.04	0.06	5.22***	0.46
Concentration of residents living in rural areas	0.00	0.02	0.52***	0.15
Concentration of residents aged 65 and over	-0.25***	0.08	-2.92***	0.62
*Healthcare capacity measures*				
Acute care hospital beds per 1,000 residents	0.01	0.00	-0.11**	0.03
PCPs per 100,000 residents	0.00	0.00	0.00	0.00
Medical specialists per 100,000 residents	0.00	0.00	-0.01***	0.00
Surgeons per 100,000 residents	0.00	0.00	0.01***	0.00
**Hospital-level**				
Number of patients	-0.00***	0.00	0.00	0.00
Number of physicians (log)	-0.09***	0.00	1.38***	0.02
Concentration of patients from outside the CBSA	0.01	0.01	0.61***	0.04
Academic hospital	0.00	0.01	-0.05*	0.03
Charlson score	0.05***	0.00	-0.01	0.01
Concentration of patients living below federal poverty line†	-0.08	0.06	1.23***	0.31
Concentration of patients with graduate education†	-0.16*	0.08	-1.02*	0.41
Concentration of patients living in a rural area †	-0.02	0.01	0.55***	0.07
Concentration of Hispanic patients †	0.19***	0.04	-0.22	0.24
Concentration of black patients	0.03	0.02	-0.62***	0.12
Constant	0.84***	0.06	-2.87***	0.51
Year fixed effects	Yes		Yes	
State fixed effects	Yes		Yes	
Hospital random effects	Yes		Yes	
Observations (N)	12179		12179	
Hospitals	3390		3390	
Log-likelihood	2407.91		-30456.60	
d. f.	74		74	

**Abbreviations:** CBSA, core based statistical area; Coeff, coefficient; d. f., degrees of freedom; HSA, hospital service area; PCP, primary care physician; S. E., standard error.

**Note:** Standard errors in parentheses;

* p < 0. 05,

** p < 0. 01,

*** p < 0. 001; two-tailed tests. Estimates are derived from random effects negative binomial (external ties) and tobit (clustering) regression models.

^†^ Estimated using levels found in patients’ home zip codes.

## Discussion

We aimed to determine whether the structure of physician referral networks that form around beneficiaries undergoing THR differ based on the racial composition of the community that those networks serve. We observed striking differences among networks. Specifically, we found that physician referral networks in communities with higher concentrations of black residents were more likely to cluster in small highly interconnected groups, and have few or no external ties than those serving communities with lower concentrations of black residents. Although a high clustering coefficient can also indicate intense collaboration and exchange of information between network physicians, the small size and the lack of external ties among networks in communities with higher concentrations of black residents suggests that these networks may be insular. These findings have potential implications on existing disparities in access to THR.

To our knowledge, our study is the first to explicitly examine the association between race and physician referral networks in joint replacement. Traditional approaches to addressing disparities in joint replacement have focused on empowering the patient to learn more about the benefits of the procedure and engage more actively with their physician and surgeon. While these efforts are important, our findings suggest that they alone may be insufficient. Since these networks cannot be recognized by patients, they represent “invisible” barriers within the patient’s healthcare environment that could hinder their access to high-quality providers and, thus, may adversely affect their outcomes. Indeed, studies on race and hospital volume have clearly shown that blacks are more likely to seek a low-volume hospital, even when a high-volume hospital is within their own community [16]. This may be plausible since the patient is referred to an orthopedic surgeon in the network to perform the surgery, and this surgeon has admitting privileges only in certain hospitals within the sphere of influence of his/her providers’ network. Although the present study did not examine the relationship between networks and access to high-volume hospitals, our findings may provide one potential explanation for these racial disparities in access to high-quality orthopedic care. Our findings for THR may be indicative of a more general problem of access to surgical care in communities with high proportions of blacks. Hollingsworth et al. (2015) conducted a similar study looking at the networks of patients undergoing coronary artery bypass grafting procedures and found very similar results (9). On the other hand, it is unclear if these networks are more “visible” to individual physicians or whether they are aware of the impact of their network on the outcomes of their patients, and what actions they would take, if they knew this information.

To date, knowledge gained from research on physician referral networks, their characteristics, and their effect on outcomes has not been put to use to enhance patient outcomes, in large part because using network analysis to detect and study physician relationships is a fairly new field of investigation [17]. Only few published studies have mapped physician networks and investigated their impact on outcomes primarily using Medicare claims; and these studies varied significantly in their definition of a network [7, 10, 11, 18–20]. However, advances in health information technology and the current efforts to redesign the U.S. healthcare system should speed up the incorporation of findings from these studies into healthcare delivery. Findings from our study, for example, hold the premise to expand the range of possible interventions that are currently available for addressing racial disparities in joint replacement, to include provider-targeted interventions. Health plans may consider network-based approaches to enhance the performance of the networks they contain and create interventions targeted at physicians to reshape their referral networks. Real time data availability should make these data-driven network-based interventions timely and readily available for health plans.

A number of limitations should be considered when interpreting the study findings. Our physician referral networks were created based on medical claims from older adults. As such, our findings may not be generalizable to younger adults, among whom a growing concentration of THRs are being performed [21]. Moreover, our physician referral networks were constructed based entirely on claims data, which ignore other channels of physician interaction. Thus, we may underestimate the number of ties (including external ones) that networks may have. Finally, we had no access to information about physician race, which may affect the referral process.

Limitations notwithstanding, we have shown for the first time that physicians caring for Medicare THR patients organized and referred in ways that differed substantially between communities with high concentrations of blacks and those with low concentrations. These differences are invisible to patients, and will significantly discount any potential benefits realized from patient-level educational interventions. Therefore, our study highlights the importance of exploring systems-level levers as complementary to patient education in addressing existing disparities in joint replacement.
